# Optimization of Process Control Parameters for Fully Mechanized Mining Face Based on ANN and GA

**DOI:** 10.1155/2021/5557831

**Published:** 2021-05-25

**Authors:** Hongze Zhao, Zhihai Xu, Qi Li, Tao Pan

**Affiliations:** ^1^School of Energy and Mining, China University of Mining and Technology (Beijing), Beijing 100083, China; ^2^State Key Laboratory for Geomechanics and Deep Underground Engineering, China University of Mining and Technology (Beijing), Beijing 100083, China; ^3^CHN Energy Information Technology Co., Ltd., Beijing 100011, China

## Abstract

In the traditional optimization mathod, the process control parameters for fully mechanized mining face are determined by experts or technicians based on their own experience, which is lack of scientific basis, and need long production adjustment cycle. It is cause large loss, and not conducive to improving mine production efficiency. In order to solve this problem, the study proposes a process control parameter optimization method based on a mixed strategy of artificial neural network and genetic algorithm and uses a cross-entropy cost function to optimize an artificial neural network, which improves the learning speed and fitting accuracy of the neural network. Using the historical production data of a fully mechanized coal mining face, taking the pulling speed of the shearer, hydraulic support moving speed, chain speed of scraper conveyor, chain speed of stage loader, emulsion pump outlet pressure, and spray pump outlet pressure as the optimization objects and taking the value range of each process control parameter as a constraint condition to establish a mixed strategy optimization model of process control parameters for a fully mechanized mining face, each process control parameter is optimized with the output of coal per minute as the optimization goal. The results show that the method has high accuracy and short optimization process time and can effectively improve the production efficiency of the working face.

## 1. Introduction

The production system of the fully mechanized mining face is the most important part of the coal mining system. The production capacity of the working face directly determines the production capacity of the mine. At present, the level of automation and intelligence of fully mechanized mining faces in our country is low, and there is a widespread problem of inadequate connection between working procedures [1]. Since the production system of the working face is a complex series system, each process affects the other; it is difficult to establish an accurate process mechanism model. In the actual production process, the process control parameters of each production equipment are set and adjusted by the technicians according to their own experience, which lacks a scientific basis and needs a long adjustment period, seriously affecting production efficiency. Therefore, the production system of fully mechanized mining face urgently needs a new intelligent optimization method of process control parameters to improve production efficiency.

Due to the late start of our country's research on the automation and intelligence of fully mechanized mining faces. At present, the research is mainly about the optimization of the performance of a single device or the optimization of the reasonable cooperation between two devices at the equipment level, such as the cutting mode recognition of the shearer [2], study on the hydraulic support displacement accuracy [3], coordinated control research on driving the sprocket of the scraper conveyor under heavy load [4], and the research on the speed coordinated control of coal shearer and scraper conveyor [5]. In theory, improving the performance of a single device can play a role in improving the production capacity of the mining face. However, the working face production system is not a parallel system, each production link affects the other. Only improving the performance of one link may limit the ability of other links, causing a “ bottlenecks” phenomenon. Therefore, the key to improving the production capacity of the working face should optimize the key process control parameters of each production equipment from the overall working face production system, so that the various production processes can operate in coordination. However, there are currently almost no researchers in the mining industry conducting research in this area.

At present, the intelligent optimization method of process control parameters represented by the genetic algorithm [6] and grey neural network [7] has been successfully applied to industrial production industries such as mechanical processing and mineral processing. For example, Devanathan et al. used the firefly algorithm to optimize the process control parameters of the friction stir welding process of metal matrix composites [8]. Li et al. used an overlimit learning machine and association rule algorithm to optimize the process control parameters of the blast furnace surface [9]. Abdullah et al. used a combination of the artificial neural network and the genetic algorithm to optimize the process control parameters of pectinase-assisted low-temperature extraction of cashew apple juice [10]. These intelligent optimization methods find laws and build models from historical production data, which solves the problem that it is difficult to build process mechanism models in complex industrial production. A large number of sensors have been installed at the production sites of many fully mechanized mining faces in our country to monitor production equipment, and a large amount of historical data closely related to the production process has been accumulated in the long-term production. These data are condensed in various undiscovered laws throughout the entire life cycle of the production process. Therefore, in view of the problem that most of the process control parameters of the current fully mechanized mining face production system are manually set and adjusted based on experience, this paper draws on the process control parameter optimization methods of other industries and proposes a data-driven modelling method. In this paper, a hybrid strategy modelling method combining improved BP neural network and genetic algorithm is used to optimize process control parameters. After field verification, the intelligent optimization method proposed in this paper reduces the time of manual decision-making and strengthens the cooperation between various processes. In the same production time, it can cut nearly two cuts of coal more than before, which improves production efficiency.

## 2. Introduction to the Production System of Fully Mechanized Mining Face

**Figure 1:**
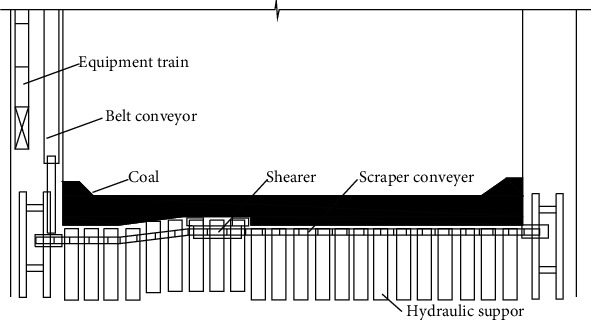
Coal mining process flow chart of fully mechanized mining face.

The coal mining process of a fully mechanized coal mining face is shown in Figure 1, which includes five major processes: coal mining, coal loading, coal transportation, support, and goaf treatment. The coal cut by the shearer passes through the scraper conveyor transported to the bottom of the working surface, after passing through the stage loader and the crusher, it is transported to the outbound system by the flat lane belt conveyor. The scraper conveyor and hydraulic support need to be matched with the shearer to maximize the production capacity of the shearer. The conveying capacity of the scraper conveyor should be greater than the production capacity of the shearer, and the moving speed of the hydraulic support should be greater than the working speed of the shearer.

**Figure 2:**
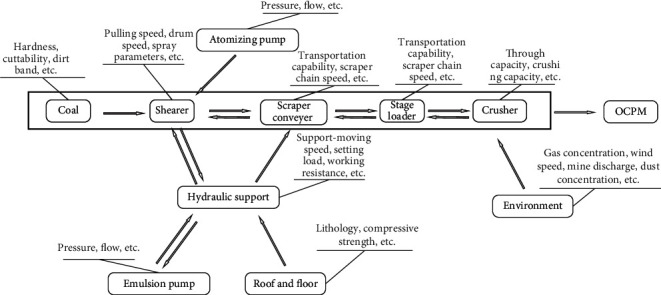
Coupling relationship diagram of the production system in fully mechanized mining face.

The fully mechanized mining face production system is a complex series system, which can be divided into three subsystems, namely, the equipment subsystem, the environment subsystem, and the coal mining process subsystem. Each process in the production process generally contains multiple process control parameters. The fluctuation of any process control parameter will have an impact on other subsequent production processes and affect the comprehensive production index of the mine. The coupling relationship between the processes and process control parameters of the fully mechanized face production system is shown in Figure 2.

**Figure 3:**
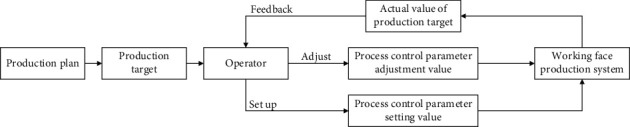
Flow chart for optimization of working face process control parameters.

In the actual production process, the process control parameter optimization process of each production equipment is shown in Figure 3. When business managers make production plans, they quantify the production plan as specific production goals, and then technicians decompose the production goals into the initial set values of the process control parameters of each production equipment based on their own experience. The equipment operator operates the equipment for production according to the set value of the process control parameter. When the actual value of the production target deviates more from the expected value, the dispatcher will notify the technician. And the technicians then adjust the process control parameters of each production equipment according to their own experience and feed them back to the equipment operators. This method of manually setting and adjusting process control parameters lacks a scientific basis. It is not conducive to improving the production efficiency of the mine, and the degree of automation is low, not only does it not give full play to the performance of the production equipment but also the production control process is easily affected by the subjective influence of the operator.

## 3. Methods and Data

### 3.1. Dataset 1 Description

This dataset is the historical production data of the key process control parameters of each production equipment collected by the sensors at the production site of the fully mechanized mining face, which are the pulling speed of shearer (PSS), hydraulic support moving speed (HSMS), chain speed of scraper conveyor (CSSC), chain speed of stage loader (CSSL), emulsion pump 1 outlet pressure (EPOP1), emulsion pump 2 outlet pressure (EPOP2), spray pump 1 outlet pressure (SPOP1), and spray pump 2 outlet pressure (SPOP2), and the comprehensive production index is the output of coal per minute (OCPM).

**Figure 4:**
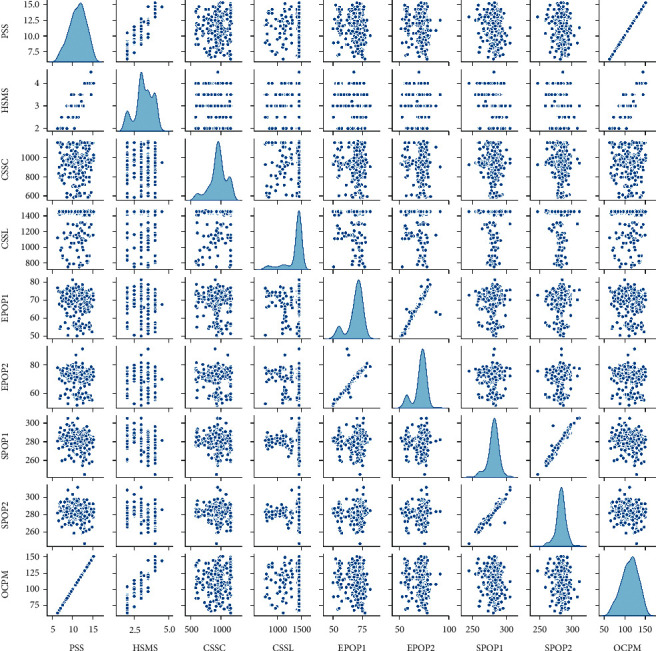
Pearson correlation analysis matrix diagram.

In this paper, the Pearson correlation analysis method is used to analyse the historical production data and preliminary analysis of the relationship between the parameters.  Figure 4 is the correlation matrix diagram of each process control parameter drawn according to the calculation results; it can be seen from the figure that there is a high linear correlation between the PSS, HSMS, and OCPM. The SPOP1 and SPOP2 are highly linearly related, and fluctuate up and down within the value range of 50–80. The EPOP1 and EPOP2 have a high linear correlation and fluctuate up and down within the range of 250–300. There is a certain degree of correlation between CSSC, CSSL, and other process control parameters, but they are not linearly related. It can be seen from the analysis results that the operating mechanism of each production equipment in the fully mechanized face production system is very complicated, and it is difficult to establish an accurate process mechanism model to quantify and establish connections between various parameters.

### 3.2. Methods

**Figure 5:**
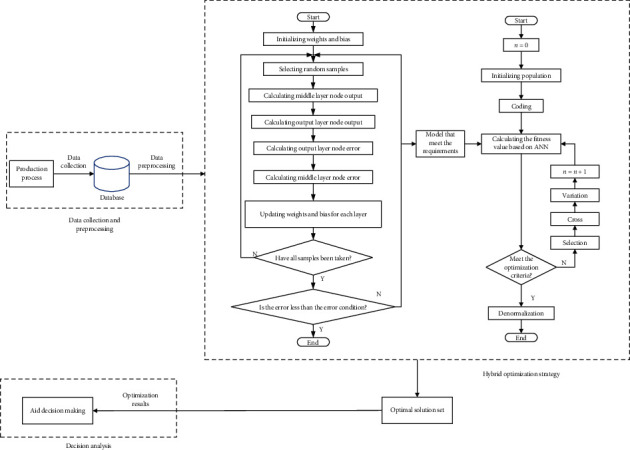
Mixed optimization process of process.

We propose a process control parameter optimization method based on a mixed strategy of artificial neural network (ANN) and genetic algorithm (GA). The method includes two core modules, namely, ANN-based process control parameter coupling relationship modelling and GA-based process control parameters optimization, and the process is shown in Figure 5. Artificial neural network [11–13] is a mathematical model created by imitating the operation of animal brain nerve cells. It has strong nonlinear processing capabilities. The genetic algorithm [14–16] is a mathematical model of optimal solution search based on biological evolution theory. The genetic algorithm based on the Pareto optimal concept [17, 18] has low complexity, good diversity of optimal solutions, and other advantages; it is very suitable for use in the field of optimization research.

#### 3.2.1. Process Control Parameter Coupling Relationship Modelling

The specific calculation steps for constructing the process control parameter model using the traditional neural network are as follows:

(1) Step 1: initialize the weights *w*_*ij*_^*l*^ and bias *b*_*j*_^*l*^, which is a random number between [−1, 1]; *l* represents the number of layers, *i* represents the *i*′*th* neuron in the *l* − 1 layer, and *j* represents the *j*′*th* neuron in the *l* layer. The following *i*, *j*, and *l* have the same meaning.
(2) Step 2: randomly select input samples *a*_*j*_^1^ and target output value *T*.
(3) Step 3: calculate the weighted input value *z*_*j*_^*l*^ and the output value *a*_*j*_^*l*^ of each neuron in a forward direction layer by layer:
  (1) $$\begin{array}{c} z_{j}^{l}=\sum_{i=1}w_{ij}^{l}a_{j}^{l-1}+b_{j}^{l},\\ a_{j}^{l}=f\left(z_{j}^{l}\right). \end{array}$$
The activation function *f* uses the Sigmoid function:*f*=1/1+*e*^−*μ*^.
(4) Step 4: calculate the mean square error between the network output value *a*^*L*^ and the target output value *T*, *L* represents the output layer:
  (2) $$\begin{array}{c} C=\frac{1}{2n}\sum_{n=1}{\left(a^{L}-T\right)}^{2}. \end{array}$$
(5) Step 5: use stochastic gradient to update the weights and bias of each layer, layer by layer.
  (3) $$\begin{array}{c} w_{ij}^{l}=w_{ij}^{l}-\mu \frac{\partial C}{\partial w_{ij}^{l}},\\ b_{ij}^{l}=b_{ij}^{l}-\mu \frac{\partial C}{\partial b_{ij}^{l}}. \end{array}$$
(6) Step 6: repeat steps 3 to 5 until the error meets the set conditions.

**Figure 6:**
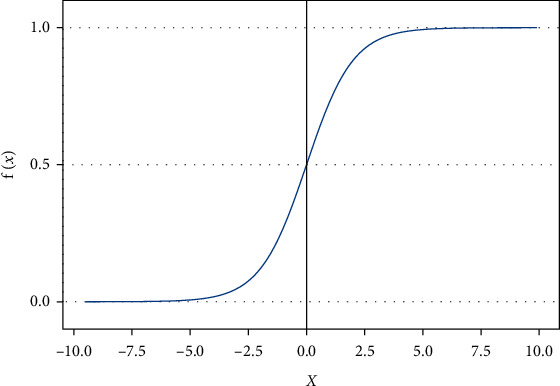
Sigmoid function image. Control parameters for coal mining face.

The traditional neural network uses the mean square error function when measuring the network output value *a*^*L*^ and the target value *T*. When the s-shaped function is used as the activation function, such as the Sigmoid function, its function image is shown in Figure 6. Using the chain rule to find the partial derivative of the weight *w* and the bias *b* for loss value *C*, as shown in equation (4) and equation (5). It can be seen from Figure 6 that when the output value of the neuron is close to 0 or 1, the curve becomes more and more smooth. As a result, *f*(*z*_*j*_^*L*^)′ becomes very small, leading to the fact that ∂*C*/∂*w* and ∂*C*/∂*b* will be very small at the same time. This change will eventually cause the learning speed of the neural network which uses gradient descent as the learning algorithm to become very slow:

(4) $$\begin{array}{c} \frac{\partial C}{\partial w}=\frac{1}{2n}\sum \left(a^{L}-T\right)f{\left(z_{j}^{L}\right)}^{\prime }x, \end{array}$$

(5) $$\begin{array}{c} \frac{\partial C}{\partial b}=\frac{1}{2n}\sum \left(a^{L}-T\right)f{\left(z_{j}^{L}\right)}^{\prime }. \end{array}$$

**Figure 7:**
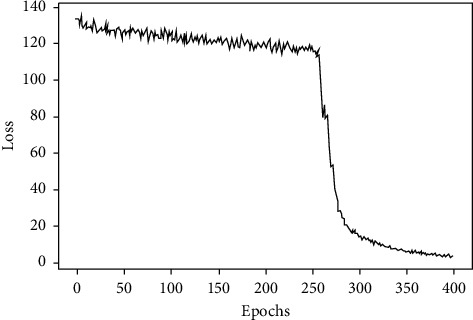
Neural network learning situation with mean square error cost function.

**Figure 8:**
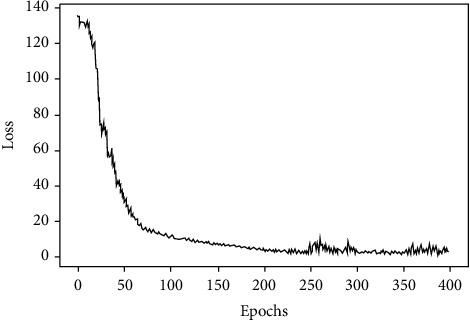
Neural network learning situation with cross-entropy cost function.

In order to solve this problem, this study uses a cross-entropy cost function to replace the traditional mean square error cost function to optimize the neural network to improve the learning speed of the neural network and its form is as equation (6). When the s-shaped function is used as the activation function, such as the Sigmoid function, using the chain rule to find the partial derivative of the weight *w* and the bias *b* for loss value *C*, as shown in equations (7) and (8), it can be found from equations (7) and (8) that *f*(*z*_*j*_^*L*^)′ generated during the chain rule of the mean square error function does not appear in the derivation result of the cross-entropy function. This change eliminates the effect that the learning speed of the neural network using gradient descent as the learning algorithm becomes very slow when the output value of the neuron is close to 0 or 1. Figures 7 and 8, respectively, show the learning situation of the neural network when the mean square error cost function and the cross-entropy cost function are used. It can be seen that the learning speed of the neural network using the mean square error cost function is very slow at the beginning, and the situation improves with the increase of the number of iterations, while the learning speed of the neural network using the cross-entropy cost function is very fast at the beginning, and it takes fewer iterations to achieve the best results:

(6) $$\begin{array}{c} C=-\frac{1}{n}\sum \left[T\;\ln\;a^{L}+\left(1-T\right)\ln\left(1-a^{L}\right)\right], \end{array}$$

(7) $$\begin{array}{c} \frac{\partial C}{\partial w}=\frac{1}{n}\sum x\left(f\left(z_{j}^{L}\right)-T\right), \end{array}$$

(8) $$\begin{array}{c} \frac{\partial C}{\partial b}=\frac{1}{n}\sum \left(f\left(z_{j}^{L}\right)-T\right). \end{array}$$

#### 3.2.2. Optimal Modelling of Process Control Parameters

After obtaining the process control parameter coupling relationship model with a better fitting effect, directly use the difference between the output value of the established neural network coupling relationship model and the target value of the optimization target as the fitness function of the genetic algorithm to optimize each process control parameter; the specific steps are as follows:

(1) Step 1: initialize the population. The population evolution generation is set to *G* = 200, the population size is set to *P* = 100, which means it contains 100 individuals, each individual contains *m* = 8 chromosomes, and each chromosome corresponds to a process parameter; the selection operator is set to 0.2, and the crossover operator adopting the analog binary crossover operator and the mutation operator is set to 1⁄m, which means that a feature of the next generation will mutate.
(2) Step 2: coding. In this study, the binary coding method is used, and each chromosome is encoded with a binary number. The representation of each individual in the population is *x*=[*x*_1_ ,  *x*_2 _,   …, *x*_*m*_].
(3) Step 3: calculate the fitness vector. The codes of all individuals in the population are sequentially input into the trained process control parameter coupling relationship model, and the difference between the output value and the target value is used as the fitness vector. The mathematical model is shown in the following equation:
  (9) $$\begin{array}{c} \left\{\begin{array}{c} \min f\left(x\right)=Y-Z,\\ s.t.X_{\min}\leq X_{i}\leq X_{\max},\;1\leq i\leq m. \end{array}\right) \end{array}$$
In the previous equation, *Y* is the output value of the process control parameter coupling relationship model, *Z* is the target value of the optimization problem, *X*_*i*_ is each process control parameter, *X*_min_ is the lower limit of each process control parameter, and *X*_max_ is the upper limit of each process control parameter.
(4) Step 4: Pareto sorting.
(5) Step 5: according to the selection operator, select the corresponding individuals from the individuals sorted by Pareto to reproduce their offspring.
(6) Step 6: according to the crossover operator, the genes between the individuals selected in the fifth step are crossed to produce the next generation of individuals.
(7) Step 7: randomly mutate the next generation of individuals according to the mutation operator.
(8) Step 8: repeat steps 3 to 7 until the result reaches the set evolution generation.
(9) Step 9: denormalize the output results, and the result is the optimized process parameter solution set.

## 4. Results and Discussion

### 4.1. Dataset 2 Description

The data in this dataset is selected from dataset 1 after data preprocessing steps to remove outliers and used to train and test the model in the paper. In this study, we use the deviation standardization method to standardize the data; the standardized data of the PSS, HSMS, CSSC, CSSL, EPOP1, EPOP2, SPOP1, and SPOP2 were used as the input of the artificial neural network; and the OCPM is used as the output of the artificial neural network. Then, establish the improved neural network-based process control parameter coupling relationship model. Dataset 2 made according to historical data is randomly divided into two parts according to the ratio of 8 : 2. 80% of the data is used as the training set and 20% of the data is used as the test set to train the process control parameter coupling relationship model.

### 4.2. Results and Discussion

**Figure 9:**
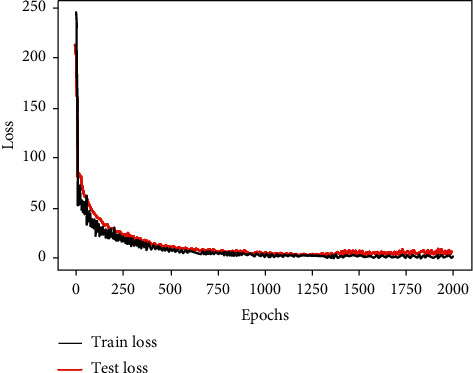
The optimized neural network error loss.

In this study, the historical production data of a fully mechanized coal mining face was used to train the process control parameter coupling relationship model based on artificial neural networks, and the mean square error was used as the model evaluation method to evaluate the prediction results of the model. The mean square error [19, 20] is used to restore the degree of square distortion, which is the average of the sum of squares of the prediction errors. It avoids the problem that positive and negative errors cannot be added. Since the error is squared, the role of the error in the indicator is strengthened, thereby improving the sensitivity of this indicator. After parameter adjustment and optimization, a neural network with 3 hidden layers is finally established and the number of nodes in each hidden layer is 6, 4, and 2 respectively. The optimized model error changes are shown in Figure 9, it can be found that the model performs very well both on the test set and training set. As the number of training iterations increases, the error finally oscillates smoothly around 0. This shows that the model has a strong generalization ability.

**Figure 10:**
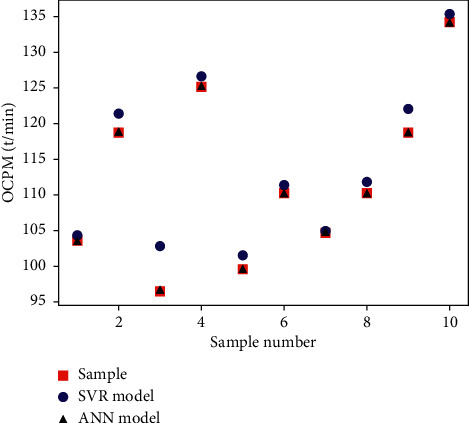
Comparison chart of forecast results.

In order to further verify the reliability and superiority of the process control parameter coupling relationship model based on the improved ANN. This study uses the same data set with the standardized data of the PSS, HSMS, CSSC, CSSL, EPOP, and SPOP as the input of the model and the OCPM as the output of the model. Establish a support vector regression- (SVR-) based [21] process control parameter coupling relationship model, and compare the prediction results of the two models.  Figure 10 is a comparison chart of prediction results. It can be seen from the figure that the predicted value of the SVR model is generally larger than the sample value, the predicted value of the improved ANN model is very close to the sample value, and the fitting effect is better. It shows that the method proposed in this study can fit the coupling relationship between process control parameters well.

In this study, the value 113 of OCPM was taken as an optimization target to analyse the process parameters optimized by the process control parameter optimization model.  Table 1 is the historical process control parameter data, and Table 2 is the process control parameter data optimized by the mixed optimization method. Comparing the results in the two tables, we can find that the optimized process parameters are very close to the process parameters in the historical data, which are in line with the actual situation and can be used to guide actual production.

**Figure 11:**
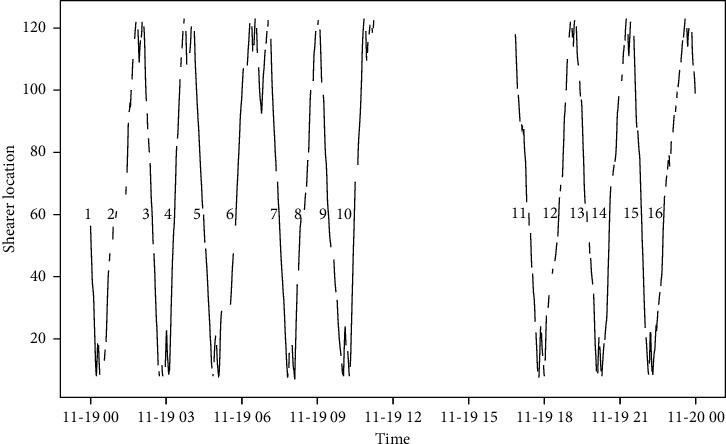
Trajectory diagram of shearer before using intelligent optimization method.

**Figure 12:**
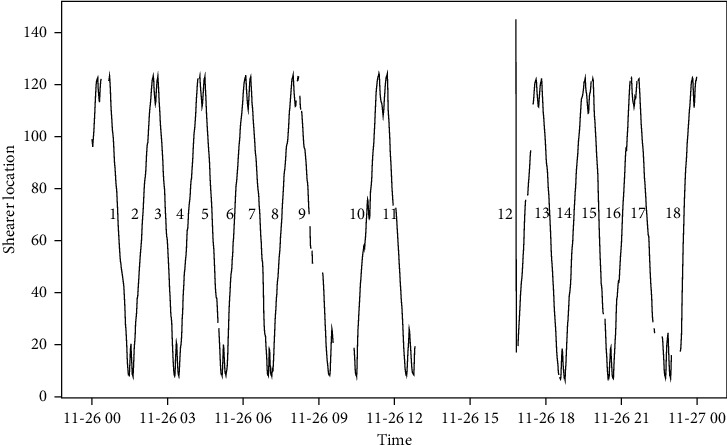
Trajectory diagram of shearer after using intelligent optimization method.

After verification in the production site of a fully mechanized mining face, the intelligent optimization method proposed in this paper reduces the time of manual decision-making, strengthens the cooperation between various procedures, and greatly improves the production efficiency of the mining face. Before using this method, the process control parameters of the mining face production site are set and adjusted manually based on experience. As shown in Figure 11, the working face production system produces 19 hours a day and cuts 16 cuts of coal every day. The average time to cut one cut of coal is about 71 minutes and the calculation process is shown in equation (10). After using the method proposed in this paper, the time of manual decision-making is reduced. As shown in Figure 12, the working face production system produces 19 hours a day and cuts 18 cuts of coal every day. The average time to cut one cut of coal is 63 minutes and the calculation process is shown in equation (11). Each cut of coal can be reduced by 8 minutes, nearly 2 cuts of coal can be cut every day, and the production efficiency has been greatly improved:

(10) $$\begin{array}{c} \frac{19\times 60}{16}=\frac{1140}{16}=71.25, \end{array}$$

(11) $$\begin{array}{c} \frac{19\times 60}{18}=\frac{1140}{18}=63.33. \end{array}$$

## 5. Conclusion

1. This paper uses the cross-entropy cost function to optimize the traditional neural network, which speeds up the learning speed of the model and reduces the number of iterations of the model. Compared with the SVR model, the improved neural network model has a higher fitting accuracy for the coupling relationship of the process control parameters, which is more suitable for establishing the nonlinear relationship between process control parameters and production goals.
2. This paper proposes a process control parameter optimization method based on a mixed strategy of ANN and GA, taking the neural network-based process control parameter coupling relationship model as the fitness function of genetic algorithm, which fully combines the nonlinear modelling capabilities of artificial neural networks and the global optimization capabilities of genetic algorithms. This method can quickly optimize the process control parameters of the fully mechanized mining face without artificial interference, which solves the problem of low efficiency of traditional optimization methods. And it was verified on the production site, which shortened the cutting time of each cut by 8 minutes and improved the production efficiency of the working face.
3. The method proposed in this study can only optimize the initial value of each process control parameter of the fully mechanized mining face. When the working environment changes, the process control parameters will also fluctuate. Therefore, future research should also add a dynamic adjustment model of process control parameters to dynamically adjust each process control parameter in response to changing working environments.

## Figures and Tables

**Table 1:** Historical data.

PSS m/min	HSMS number/min	CSSC r/min	CSSL r/min	EPOP1 0.1 MPa	EPOP2 0.1 MPa	SPOP1 0.1 MPa	SPOP2 0.1 MPa
11.56	3.34	1099.92	1448.91	59.64	61.59	293.36	297.04
11.56	3.34	1099.69	1449.08	54.06	56.46	292.46	296.60
11.56	3.34	1099.34	1449.77	59.67	61.87	291.88	296.61
11.56	3.34	1099.92	1449.78	55.96	57.74	292.46	299.18
11.56	3.34	1099.54	1450.00	55.26	57.43	293.82	297.13
11.56	3.34	1099.58	1449.84	54.62	56.63	292.93	297.89
11.56	3.34	1099.52	1449.89	52.49	54.15	294.15	300.57

**Table 2:** Optimized data.

PSS m/min	HSMS number/min	CSSC r/min	CSSL r/min	EPOP1 0.1 MPa	EPOP2 0.1 MPa	SPOP1 0.1 MPa	SPOP2 0.1 MPa
11.57	3.20	1099.89	1449.43	56.92	54.61	297.58	297.08
11.57	3.28	1099.79	1449.63	56.57	54.61	294.40	297.08
11.57	3.42	1099.80	1449.62	56.57	54.37	294.40	297.08

## Data Availability

The data used to support the findings of this study are shown in Supplementary Materials.

## Supplementary Materials

Supplementary Materials The three supplementary data files provided in this paper are obtained by our laboratory. Data 1 is dataset 1 used in the paper; it contains all the historical production data of the key process control parameters of each production equipment collected by the sensors at the production site of the fully mechanized mining face. Data 2 is dataset 2 used in the paper which is filtered from dataset 1 to train and test the model in the paper. Data 3 is the operating trajectory data of the shearer.
